# Feasibility and Challenges of Performing Magnetoencephalography Experiments in Children With Arthrogryposis Multiplex Congenita

**DOI:** 10.3389/fped.2021.626734

**Published:** 2021-10-04

**Authors:** Semyon A. Golosheykin, Evgueni D. Blagoveschenskiy, Olga E. Agranovich, Maria A. Nazarova, Vadim V. Nikulin, Olesya E. Moiseenko, Russell W. Chan, Anna N. Shestakova

**Affiliations:** ^1^Center for Cognition and Decision Making, National Research University Higher School of Economics, Moscow, Russia; ^2^G.I. Turner Scientific Research Institute for Children's Orthopaedics, Ministry of Health of Russia, Saint Petersburg, Russia; ^3^Federal State Budgetary Institution ≪Federal Center of Brain Research and Neurotechnologies≫ of the Federal Medical Biological Agency, Moscow, Russia; ^4^Department of Neurology, Max Planck Institute for Human Cognitive and Brain Sciences, Leipzig, Germany; ^5^Department of Cognitive Psychology and Ergonomics, University of Twente, Enschede, Netherlands

**Keywords:** arthrogryposis, movement disorders, upper limbs, magnetoencephalography, movement-evoked fields, child, muscle transfer

## Abstract

Arthrogryposis multiplex congenita (AMC) has recently drawn substantial attention from researchers and clinicians. New effective surgical and physiotherapeutic methods have been developed to improve the quality of life of patients with AMC. While it is clear that all these interventions should strongly rely on the plastic reorganization of the central nervous system, almost no studies have investigated this topic. The present study demonstrates the feasibility of using magnetoencephalography (MEG) to investigate brain activity in young AMC patients. We also outlined the general challenges and limitations of electrophysiological investigations on patients with arthrogryposis. We conducted MEG recordings using a 306-channel Elekta Neuromag VectorView system during a cued motor task performance in four patients with arthrogryposis, five normally developed children, and five control adults. Following the voice command of the experimenter, each subject was asked to bring their hand toward their mouth to imitate the self-feeding process. Two patients had latissimus dorsi transferred to the biceps brachii position, one patient had a pectoralis major transferred to the biceps brachii position, and one patient had no elbow flexion restoration surgery before the MEG investigation. Three patients who had undergone autotransplantation prior to the MEG investigation demonstrated activation in the sensorimotor area contralateral to the elbow flexion movement similar to the healthy controls. One patient who was recorded before the surgery demonstrated subjectively weak distributed bilateral activation during both left and right elbow flexion. Visual inspection of MEG data suggested that neural activity associated with motor performance was less pronounced and more widely distributed across the cortical areas of patients than of healthy control subjects. In general, our results could serve as a proof of principle in terms of the application of MEG in studies on cortical activity in patients with AMC. Reported trends might be consistent with the idea that prolonged motor deficits are associated with more difficult neuronal recruitment and the spatial heterogeneity of neuronal sources, most likely reflecting compensatory neuronal mechanisms. On the practical side, MEG could be a valuable technique for investigating the neurodynamics of patients with AMC as a function of postoperative abilitation.

## Introduction

Arthrogryposis is a group of disorders characterized by multiple congenital contractures that affect two or more body segments. It is usually classified into three major groups: (1) amyoplasia, which is characterized by severe joint contractures, muscle weakness, and even the complete absence of some muscles; (2) distal arthrogryposis, which is a relatively milder condition mostly involving the hands and feet; and (3) the syndromic group, which combines congenital contractures with primary neurological or muscle diseases (1).

Amyoplasia, the main form of arthrogryposis multiplex congenita (AMC), has a frequency of 1 in 10,000 (2). The upper extremities are commonly involved in patients with amyoplasia, with ~56% of patients having both upper and lower extremity involvement and 17% with only upper extremity involvement (3). The upper extremities have typical deformities: the shoulder joints are held in adduction, the elbow joints in extension (less often in flexion), the wrists in flexion, the thumbs adducted, and the finger joints in varying degrees of flexion. Notably, the upper limb deformities observed in arthrogryposis conditions make performing daily activities difficult. Extension contracture is the most frequent type of elbow deformity (4). In this case, the triceps are usually strong, while the biceps and brachialis are weak or absent (5). This condition is a severe disability, especially in patients with bilateral extension contracture. Basheer et al. (6) estimated upper limb function in children with obstetric brachial plexus palsies and found that each joint affected a percentage of the whole limb function; for instance, elbow injuries decreased upper extremity function by 30%. Active elbow flexion is important in daily activity, especially for self-feeding, because it allows the hand to move to the mouth. Therefore, restoring active elbow flexion is a main task in treating children with arthrogryposis.

Active elbow flexion in patients with AMC can be restored by various surgical techniques, including long-head triceps transfer, latissimus dorsi transfer, pectoralis transfer, and free gracilis transfer (3, 7–9). Choosing a donor muscle in patients with arthrogryposis is difficult. The donor muscle must have enough strength and be suitable for transfer, and every patient must be evaluated individually, as the presence of potential donor muscles for restoring active elbow flexion is highly variable in these patients (3).

A main complication of muscle transfer is the initial inability of the patient to control a new elbow flexion movement using a transplanted muscle (e.g., the latissimus dorsi muscle) previously associated with a different movement. Acquiring the ability to move a limb that was never functional is called “abilitation.” The top-down control of a new movement is likely initiated at the cortical level, including the premotor cortex, supplementary motor cortex, and primary motor cortex. In this regard, monitoring cortical function and its plasticity is pivotal for the understanding of movement control in AMC and for the development of rehabilitation strategies. Non-invasive neuroimaging, such as functional magnetic resonance imaging (fMRI), high-density electroencephalography (hdEEG), and magnetoencephalography (MEG), has enabled significant progress in the functional mapping of the sensorimotor cortex in both healthy and clinical populations (10–13). Although fMRI is undoubtedly the most popular tool in almost all contemporary brain-related studies and EEG is a rather inexpensive, well-established method, we are confident that MEG will provide a number of benefits beyond these methods. The fMRI study environment is noisy and might intimidate young patients (14), and children are more prone to move during testing, which is highly undesirable for fMRI. In turn, hdEEG requires a long preparation period of applying EEG electrodes and adjusting them to ensure adequate impedance. The MEG approach, which does not have these disadvantages, outperforms EEG in spatial resolution and fMRI in temporal resolution, making it the most suitable for neuromonitoring patients with arthrogryposis.

MEG is an efficient approach to somatotopically map the human sensorimotor cortex (15). Reliable results have been achieved with even relatively low-density biomagnetometer arrays (16–20). Later, great success was achieved in studying slow premovement motor fields (MFs) and movement-evoked fields I and II (MEFIs and MEFIIs) related to voluntary finger movement (21, 22). Utilizing the powerful tool of beamforming analysis, the authors accurately measured the chronological benchmarks of voluntary movement cortical activation and proposed sustainable mechanisms for a multiphase response. According to their model, the sources of activity generating a first slow wave of MFs are allocated in the primary and partially in the secondary motor cortex and are responsible for preparing and initiating the movement. Index finger movement, for instance, was detected 40 ms before movement onset. The second phase corresponding to MEFI involves somatosensory areas and reflects proprioceptive and tactile reafferent feedback. Finally, the third component of MEFII is a complex activation pattern involving both motor and somatosensory cortices.

Event-related field patterns, like the MFs and MEFs observed in adults, have been reported in 4-year-old children (14), though they were delayed and had inverted polarities. The modulation of mu (8–12 Hz) and beta (15–30 Hz) oscillations had stronger frequency band coupling. The post-movement beta rebound (PMBR) increased with age from young children (4–6 years old) to adolescents (11–13 years old) to adults (24–42 years old) performing voluntary finger movements. Age also correlated with a significant shift from bilateral to ipsilateral representation of PMBR (23).

Considerably less attention has been paid to MEG studies of cortical activation associated with proximal muscles. Stephen et al. (24), however, had promising results producing and analyzing MEG waveform patterns and the dipoles of source activity during voluntary movement engaging the biceps brachii and the somatosensory stimulation of the deltoid muscle. The brain waveforms were demonstrated mostly over the contralateral-to-movement cortical areas, and the reconstructed dipoles of the activity source fit the somatotopic model of the primary motor cortex well. The accurate brain mapping of brachii muscles in patients with AMC might help estimating their individual potentials and choose suitable donor muscles for reconstructing active elbow flexion. After such operations, patients with AMC can often self-feed and are more independent in daily activities.

Since we know little about the cortical organization of muscle representation in patients with AMC, determining which parts of the complex mechanism of voluntary movement control are affected in these patients is extremely important. Most previous MEG studies of upper extremity motor function were restricted to hand and finger functions, with few attempts made to use MEG to map proximal muscle cortical representations. Most of the relevant findings mentioned above were also obtained with groups of adult subjects.

To fill this gap, we developed a paradigm of self-paced hand movement that would best suit the young AMC patients and applied it in an MEG multicase study. We aimed to demonstrate the feasibility of MEG mapping the neurodynamics of cortical responses associated with proximal muscle activity. We hypothesized that in addition to the magnitude of the MF and MEF responses, the temporal parameters of motor-related activity of patients with AMC could be deviating from those of healthy children and adult controls. We also searched for neural markers that best signify the AMC.

## Materials and Methods

### Participants

Four patients with AMC (two male and two females, 5–9 years old) who underwent reconstructive surgery at the Turner Scientific Research Institute for Children's Orthopedics, St. Petersburg, Russian Federation, were selected by a leading surgeon to participate in this study. Before operation, all patients had typical amyoplastic upper limb deformities which precluded active movement in the elbow, which in turn caused severe disabilities. All four patients had wrist contractures which were operated on prior to the MEG investigation. Three of the four patients (one male) underwent different donor muscle transfers to the biceps brachii to restore active elbow flexion (Table 1). Five normally developed children (three males, 7–10 years old) and five adults (three males, 19–38 years old) were recruited from the general population as the control group. All young participants were school children except of one AMC (age 5) who was in preschool. The adult group consisted of three college students and two college graduates. The elbow joint efficiency of an AMC patient was evaluated prior to investigation using a modified van Heest scale (5, 8). This scale includes estimation of elbow active flexion, muscle strength, and activities of daily living (ADL) and uses adaptive mechanisms for elbow flexion (table push, trunk thrust, or cervical bending). The specific criteria of the scale are summarized in Supplementary Table 1.

**Table 1:** Demographic information and clinical history of patients.

**ID**	**Gender**	**Age: years (months)**	**Elbow joint efficiency^**a**^**	**Hand**	**Elimination of the wrist contracture**	**Time after wrist surgery: years (months)**	**Restoration of elbow flexion function**	**Time after elbow surgery: years (months)**
P1	Female	9 (6)	Good	Left	Transposition m. flexor carpi radialis to m. extensor carpi radialis longus	4 (10)	Latissimus dorsi transfer to biceps brachii position	6 (9)
			Good	Right	Transposition m. flexor carpi radialis to m. extensor carpi radialis longus	5 (11)	Latissimus dorsi transfer to biceps brachii position	6 (3)
P2	Female	8 (2)	Poor	Left	Transposition m. flexor carpi radialis to m. extensor carpi radialis longus	4 (9)	Pectoralis major transfer to biceps brachii position	4 (10)
			Satisfactory	Right	Transposition m. flexor carpi radialis to m. extensor carpi radialis longus	4 (5)	Pectoralis major transfer to biceps brachii position	5 (3)
P3	Male	9 (1)	Poor	Left	N/A	N/A	N/A	N/A
			Poor	Right	Transposition m. flexor carpi radialis to m. extensor carpi radialis longus	0 (10)	N/A	N/A
P4	Male	5	Good	Left	Not required	N/A	Latissimus dorsi transfer to biceps brachii position	2 (2)
			Good	Right	Not required	N/A	Latissimus dorsi transfer to biceps brachii position	3 (8)

a *Elbow joint efficiency was evaluated prior to investigation using a modified van Heest scale (5, 8)*.

### Experimental Procedure

The experiment was carried out in accordance with the recommendations of the Declaration of Helsinki and its amendments, and the protocol was approved by the ethics committee of the National Research University Higher School of Economics. All subjects gave written informed consent in accordance with the Declaration of Helsinki.

**Figure 1:**
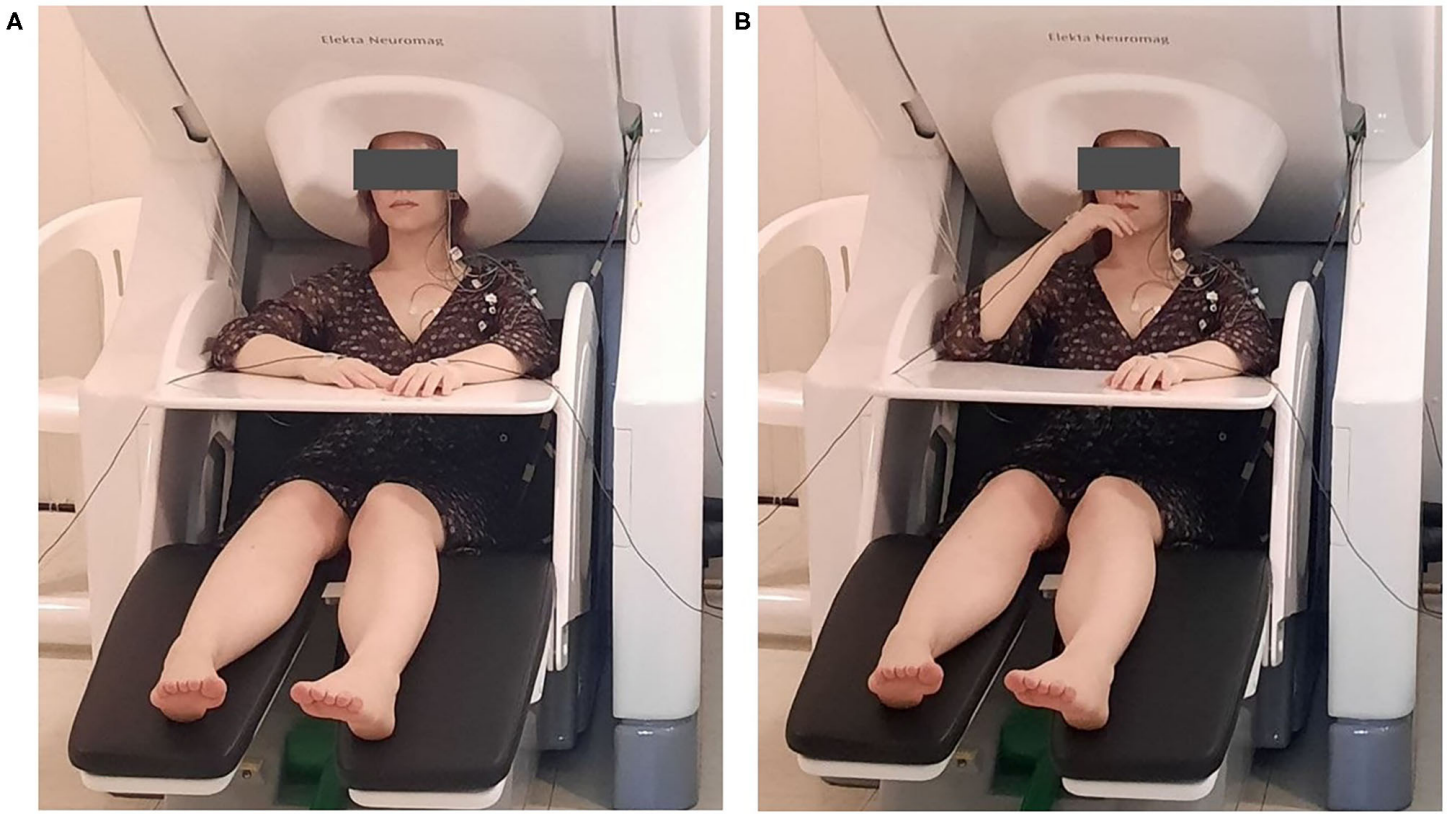
A patient performing a right elbow flexion task in the Elekta Neuromag system. The task of the patient was to bring their hand toward their mouth, imitating self-feeding, after the voice command of the experimenter. **(A)** Initial position. **(B)** Hand lifted to the mouth.

Each participant sat upright in an adjustable chair with their eyes open. Both arms rested comfortably on a plastic desk affixed to the chair (Figure 1A). Each participant was asked to bring their hand toward their mouth after a voice command of the experimenter (Figure 1B). The movement was supposed to be performed at a natural, comfortable pace to the best of the ability of the subject and imitate the self-feeding process. Each subject was asked to perform around 40 consecutive elbow flexions with each arm. The experimenter was monitoring the subject responses using on-line accelerometer and EMG channel readings as well as video surveillance camera picture. If the experimenter considered some trials unsuccessful, a few more cues were given in order to accumulate 40 trials of satisfactory quality for analysis. The subsequent verbal cues were given with the interval of ~4–10 s, but never earlier than a previous response movement had been completed. The initial left- or right-side movements were counterbalanced between subjects.

### Data Collection

The MEG data were collected using a 306-channel VectorView system (Elekta Neuromag, Finland). The 306 sensors of the VectorView system have 102 magnetometers that measure the Bz component, which is perpendicular to the surface of the detector of the magnetic field, providing widespread sensitivity, and two sets of 102 planar gradiometers that measure the gradient (∂Bz/∂x and ∂Bz/∂y), providing high focal sensitivity. The sensors are arranged in triple-sensor elements, each comprising two orthogonal planar gradiometers and one magnetometer in the same plane. We conducted MEG recordings at a sampling rate of 1,000 Hz. The hardware filters were set to AD-330 Hz. Four additional bipolar channels were used to conduct vertical electrooculogram (vEOG, left eye), electrocardiogram (ECG), and electromyogram (EMG) recordings. EMG electrodes were placed so that the active electrode was in the middle of the biceps brachii muscle. The T1-weighted images (MP-RAGE, slice = 1 mm) were obtained with a clinical, whole-body 1.5-T magnetic resonance (MR) scanner Optima MR360 Advance (General Electric, Boston, USA). A small, low-power three-axis ±3 g iMEMS ADXL330 Accelerometer (Analog Devices, Wilmington, USA) was also used. Accelerometer sensors were attached to the back of each hand by medical adhesive tape (Supplementary Figure 1).

The three-dimensional (3D) Fastrak digitizer (Polhemus, Inc., Colchester, USA) was used to digitize the positions of the head position indicator coils, the landmarks of nasion, the left and right preauricular points, and around 160 additional manually selected points to obtain information about the head shape for more accurate alignment with the anatomical MR images. Two head position indicator coils were placed above the left and right eyes, as close as possible to the hairline, while another two were placed on the left and right mastoids, respectively.

### Data Analysis

The raw continuous MEG recordings were visually inspected for artifacts using the Elekta Graph view application. The temporal signal space separation (tSSS) procedure was performed using an Elekta MaxFilter^TM^ (v2.2) to suppress magnetic interference from inside and outside the sensor array to reduce measurement artifacts and transform data between different head positions. All further preprocessing steps and data analysis were done using the Brainstorm software package (v3.210624) (25), which is documented and freely available for download online under a GNU public license (http://neuroimage.usc.edu/brainstorm).

The MRI volume was processed in the FreeSurfer software package with “recon-all” shell script. An automatic segmentation and artifact correction were performed. The individual anatomical model was co-registered with functional MEG data using 15,000 vertices of the cortical surface as a default. The landmarks of nasion, left and right preauricular points, anterior/posterior commissures, and interhemispheric points were used as fiducial points. Additional fine tuning was done if needed with 150 head points previously modeled by the Polyphemus system.

The ECG and blink artifacts were detected and corrected using the signal-space projection (SSP) protocol on a continuous MEG. Movement-onset events were then visually determined using the deflection of 3D accelerometer waveforms. Trials with no identifiable movement trajectory and/or noticeable EMG activity on contralateral hand muscles were excluded from further analysis. Trials with a response onset asynchrony (ROA) of <5 s were excluded from further analysis. The MEG channel data were filtered with a 0.03–30-Hz bandpass and an EMG with a highpass of 10 Hz. The 2,500-ms epochs were created with a zero time value at the movement onset, with a −1,500- to −500-ms reference baseline and 1,000-ms-long poststimulus intervals. An average of 41 ± 5 epochs were obtained to calculate the event-related magnetic field (ERF) per condition for each subject. Since individual brain morphology varies and the positioning of the undersized head of a child in an adult sensor array can vary considerably, for illustration purposes in sensor space, we subjectively selected one representative sensor per hemisphere per participant. The following criteria were used: (1) the magnetometer channel located over or near the cortical areas previously known to be related to motor performance, (2) a maximum amplitude of the magnetic field 0–500 ms from the detected movement onset, and (3) given several channels showing relatively similar maximum amplitudes in a window of interest, the one with the lowest noise and artifact distortion. For reference purposes, the AMC magnetometer data have been complemented with the gradiometer signal traces retrieved from the same sensor clusters (Supplementary Figure 2).

We used the linearly constrained minimum variance (LCMV) beamformer algorithm to estimate the sources of movement performance. Considering the superficial locations of the regions of interest in our study and anticipating relatively focal activation effects corresponding to proximal muscle engagement, we used only planar gradiometer data to perform beamformer analysis. For forward modeling, we used the overlapping spheres approach with the cortex as source space. Noise matrices were calculated from individual trial baseline −1,500 to −500 ms before registered movement onset. Covariance matrices were calculated in 0–1,000 ms intervals. The data covariance regularization was median eigenvalue. The main estimated value was the pseudoneural activity index (PNAI), which is a modified version of the neural activity index introduced by van Veen et al. (25–27) and is analogous to *z*-scoring. Finally, FreeSurfer Desikan–Killiany cortical parcellation was used to classify activity regions (28).

For the purpose of visualization on the 3D cortex model, unconstrained sources consisting of three dipoles with orthogonal orientations were collapsed using the norm of the vectorial sum of the three orientations at each vertex: S = sqrt(Sx2 + Sy2 + Sz2).

The power of the acceleration time series was used to visualize hand movement results. The three movement axes collapsed into the single cluster for each hand by calculating total power, the linear sum of squared values.

Because it was found difficult to maintain consistent performance using standardized pre-recorded verbal cues in patients with AMC, the experimenter had to give live verbal cues. Therefore, the beginning of a trial was decided to associate with the movement onset initiation which is technically the response of the subject to a cue. The ROA was assessed separately for the left and right hands of every participant as the time between two consequent elbow flexion movement onsets. Occasionally, the experimenter had to repeat instructions or give a comment on a performance, so a few longer intervals between trials had place during the run. The values of ROA which were greater than 3SD were excluded from averaging.

We selected the magnetometer sensors using the optimal signal-to-noise ratio (SNR) criteria: minimal noise and maximal signal observed above motor cortical areas. Both types of sensors are sensitive to motion artifacts and have their advantages and disadvantages (29) in terms of SNR and depth sensitivity. For example, gradiometers demonstrate better SNR, but a weaker signal. Using magnetometers, one can observe stronger motor responses as compared with gradiometers. Therefore, we selected magnetometers for the demonstration of signal traces while performing source analysis using gradiometers.

## Results

### AMC Patients

#### Patient #1 (P1)—Girl, 9 Years Old

P1 had a latissimus dorsi transfer to the biceps brachii 6 years before the investigation. Her elbow joint efficiency was good on both sides. See Table 1 for clinical history details.

**Figure 2:**
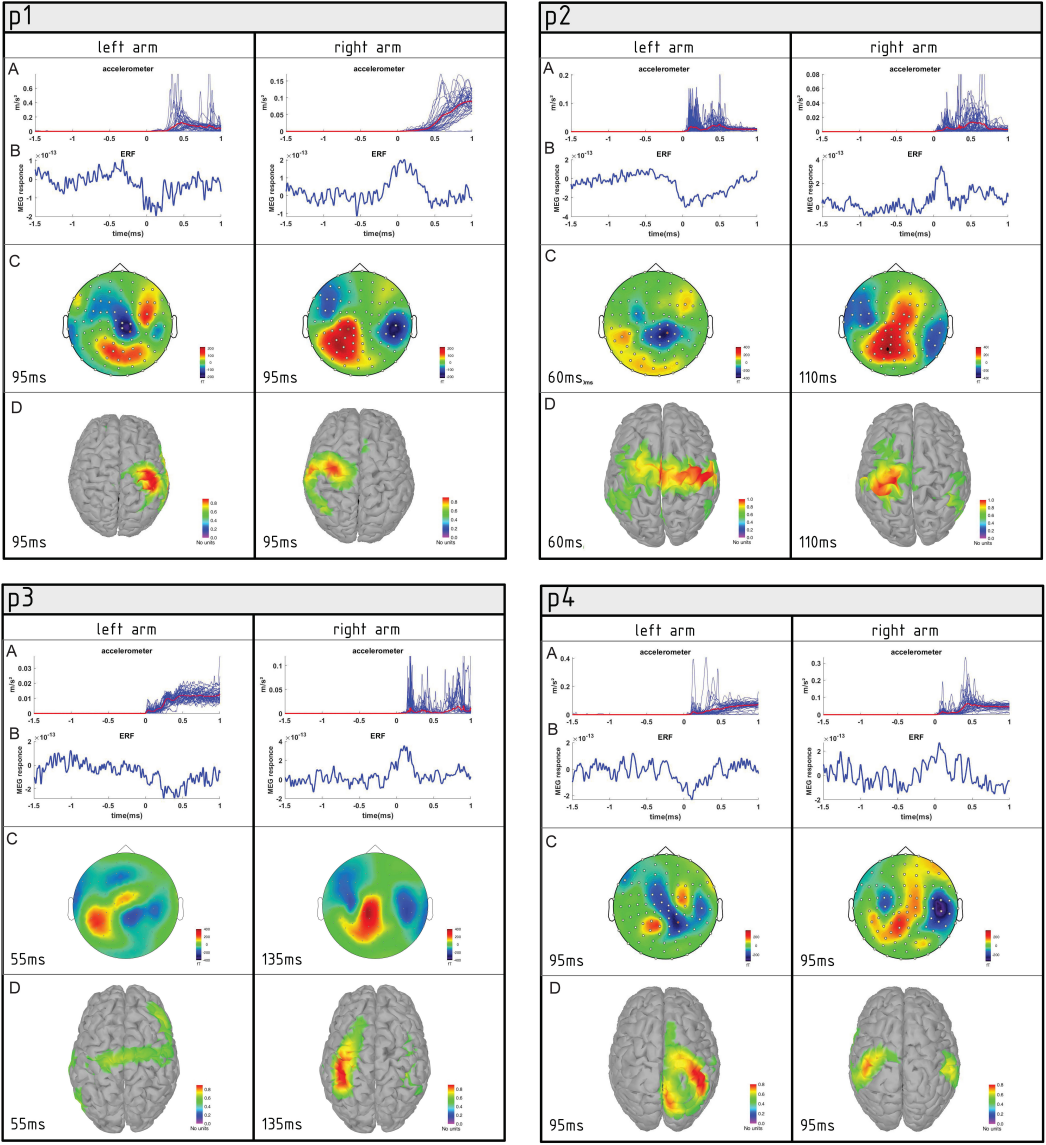
Summary of movement-associated activity in four AMC patients (P1, P2, P3, and P4) performing semi-self-paced elbow flexion. **(A)** Plot of individual accelerometer power trials overlaid for the left and right elbows. **(B)** Averaged event-related fields (ERFs) at the representative channel. **(C)** Topographies of averaged ERFs at maximum peak latency. Positive values indicate magnetic flux exiting the head (red colors) and negative flux entering the head (blue colors). **(D)** Source reconstruction of ERFs. Neural activity is superimposed on the individual cortex model.

The movement duration counted as uplift moment to return to starting position took 1.3 s for the left hand and 2.1 s for the right hand. The left-hand movements had more consistent trajectory across the trials than the right side (Figure 1 and Supplementary Table 2). Event-related magnetic fields had a prominent maximum ~95 ms from the movement onset in the hemisphere contralateral to the moving hand. The amplitude was almost the same in both conditions (Table 2), but the rising slope of MEF associated with left-hand movement was higher than for the right. This goes hand in hand with a difference in accelerometer trace trajectories. The right-hand movement was reflected in the left hemisphere (LH) by wider but less distorted magnetic field deflection. The source of neural activity was more focused, and a higher-amplitude brain response was observed in the right hemisphere (RH) during the left-hand movement that covered the parts of the precentral and postcentral gyri that supposedly represent hand control as well as a part of the superior parietal lobule. The right-hand movement was associated with more dispersed sources of activity in the LH involving parts of precentral, postcentral, and supramarginal gyri (Table 2). According to these results, although both hands earned the highest evaluation score of 5, the left and right elbows were functionally slightly different at the moment of investigation (Figure 2).

**Table 2:** Descriptive information about individual subject and group averaged brain 800 responses associated with the semi-voluntary elbow flexion task performance: latencies (Lat) (ms); peak amplitudes (Amp) (fT), of MF, MEFI, and MEFII responses measured at the representative channel (MEG chan.); names of the brain regions associated with underlying source activities. Groups: AMC, AMC patients; HCC, healthy control children; HCA, healthy control adults.

	**ID**	**Age**	**MF**		**Source activity at MF max. latency**	**MEFI**		**Source activity at MEFI max. latency**	**MEFII**		**Source activity at MEFII max. latency**	**MEG chan**.
			**Lat (ms)**	**Amp (fT)**	**Regions**	**Lat (ms)**	**Amp (fT)**	**Regions**	**Lat (ms)**	**Amp (fT)**	**Regions**	
Left elbow flexion	P1	10	−68	10	Precentral L, postcentral L, precentral R	127	−198	Precentral R, postcentral R, superiorparietal R	240	0	Precentral L, postcentral L, postcentral R	2211
	P2	8	−118	147	Postcentral R, supramarginal R, superiorfrontal L	47	−305	Precentral R, postcentral R, supramarginal R	200	43	Precentral R	2241
	P3	9	−79	0	NaN	56	−170	Precentral R, postcentral R, precentral L, postcentral L	167	−48	Postcentral R, supramarginal R	2211
	P4	5	35	−45	Precentral R, postcentral R, supramarginal R	92	−197	Postcentral R, superiorparietal R, inferiorparietal R	208	112	Postcentral R, superiorparietal R	2211
	Avg AMC		−100	0	Precentral L, postcentral L, caudalmiddlefrontal L	85	−156	Postcentral R, precentral R, supramarginal R	164	−78	Postcentral R, precentral R, supramarginal R	2211
	C1	10	−120	18	Supramarginal R, precentral R, postcentral R	40	−210	Precentral R, postcentral R	176	99	Precentral R, postcentral R, caudalmiddlefrontal R	2241
	C2	9	−141	74	Precentral R, caudalmiddlefrontal R, superiorfrontal R	97	−481	Precentral R, postcentral R	185	13	Precentral L, postcentral L, supramarginal L	0731
	C3	8	−32	25	Postcentral L, precentral L	91	−275	Precentral R, postcentral R	152	−166	Precentral R, postcentral R, caudalmiddlefrontal R	0731
	C4	10	−210	21	Precentral R, postcentral R, supramarginal R, supramarginal L	68	−454	Precentral R, postcentral R, supramarginal R	264	−44	NaN	0731
	C5	10	−233	36	Postcentral R, precentral R, supramarginal R	45	−494	Postcentral R, precentral R, supramarginal R	174	122	Supramarginal R	0731
	Avg HCC		−120	−26	Precentral R, caudalmiddlefrontal R	80	−334	Precentral R, postcentral R	176	−19	Precentral R, postcentral R, caudalmiddlefrontal R	0731
	C6	29	−99	−29	Postcentral L, precentral L	74	−329	Precentral R, postcentral R, superiorfrontal R	188	44	Precentral R, postcentral R, supramarginal R	0731
	C7	35	−26	103	Precentral R	87	−340	Postcentral R, precentral R, supramarginal R	200	15	Postcentral R, superiorparietal L	1141
	C8	38	13	38	Postcentral L, precentral L	91	−278	Postcentral R, supramarginal R	205	155	Precentral R, postcentral R	0731
	C9	22	−67	32	NaN	86	−246	Precentral R, postcentral R	248	167	Postcentral R, supramarginal R	2211
	C10	22	−102	108	Precentral R, postcentral R, supramarginal R	25	−313	Postcentral R, precentral R, supramarginal R	197	108	Precentral R, postcentral R	2211
	Avg HCA		−65	−5	Precentral R, postcentral R	88	−235	Postcentral R, supramarginal R, precentral R	200	36	Precentral R, postcentral R, supramarginal R	0731
Right elbow flexion	P1	10	−255	−30	Caudalmiddlefrontal L, postcentral L	90	203	Postcentral L, precentral L, supramarginal L	460	−72	Precentral R, postcentral R, precentral L, postcentral L	1821
	P2	8	−175	−60	Postcentral L, precentral L, supramarginal L, superiorparietal L	100	356	Postcentral L, precentral L, supramarginal L	240	−18	Precentral R	0741
	P3	9	−180	−90	Postcentral L, precentral L, superiorparietal L	136	405	Precentral L, postcentral L, supramarginal L	318	−50	Postcentral L, precentral L, supramarginal L	0741
	P4	5	−205	−60	Inferiorparietal L, supramarginal L, postcentral L, precentral L	60	270	Supramarginal R, inferiorparietal L, supramarginal L, postcentral L, precentral L	180	10	Inferiorparietal L, supramarginal L, superiorparietal L	1831
	Avg AMC		−215	−54	Precentral L, caudalmiddlefrontal L, superiorfrontal L	98	247	Precentral L, postcentral L, supramarginal L supramarginal R	253	−31	Precentral R, caudalmiddlefrontal R	0741
	C1	10	−194	−36	Caudalmiddlefrontal R, precentral R, postcentral R	28	388	Precentral L, postcentral L, caudalmiddlefrontal L	163	71	Postcentral R, superiorparietal R	2011
	C2	9	−70	−15	Caudalmiddlefrontal L, precentral R, postcentral R, superiorparietal R	88	337	Postcentral L, supramarginal L, superiorparietal L	237	−112	Postcentral L, precentral L	0741
	C3	8	−97	−10	Precentral L, postcentral L, caudalmiddlefrontal L, superiorfrontal L	72	424	Precentral L, caudalmiddlefrontal L, postcentral L	165	66	Postcentral L, precentral L	0741
	C4	10	−74	−42	Precentral L, postcentral L	63	136	Precentral L, postcentral L	129	−147	Superiorparietal R, inferiorparietal R	0741
	C5	10	−188	−67	Supramarginal L	54	445	Superiorfrontal L, precentral L, postcentral L	146	−33	Superiorfrontal L, precentral L, supramarginal R	0741
	Avg HCC		−152	11	Supramarginal L, precentral R, superiorfrontal R	54	292	Precentral L, postcentral L	182	20	Precentral L, postcentral L, supramarginal L	0741
	C6	29	33	−60	Superiorparietal L	91	115	Precentral L, postcentral L, superiorparietal L	186	−502	Precentral L, postcentral L, superiorparietal L	0741
	C7	35	−233	−54	Precentral L, postcentral L, superiorparietal L	77	394	Postcentral L, precentral L	239	−165	Postcentral L, superiorparietal L	0741
	C8	38	−5	−200	Precentral L, caudalmiddlefrontal L, superiorfrontal L	90	206	Postcentral L, precentral L	194	−210	Precentral L, postcentral L, superiorparietal L, supramarginal L	0711
	C9	22	−100	−35	NaN	48	212	Precentral L, postcentral L	210	−125	Postcentral L, supramarginal L	0741
	C10	22	−147	−78	Precentral L, postcentral L, superiorparietal L	45	235	Precentral L, postcentral L	205	−88	Precentral L, postcentral L, superiorfrontal L	0741
	Avg HCA		−152	−14	Precentral L, postcentral L, superiorparietal L	86	208	Postcentral L, precentral L	196	−180	Superiorparietal L, supramarginal L, precentral L, postcentral L	0741
Right elbow flexion	P1	10	−255	−30	Caudalmiddlefrontal L, postcentral L	90	203	Postcentral L, precentral L, supramarginal L	460	−72	Precentral R, postcentral R, precentral L, postcentral L	1821
	P2	8	−175	−60	Postcentral L, precentral L, supramarginal L, superiorparietal L	100	356	Postcentral L, precentral L, supramarginal L	240	−18	Precentral R	0741
	P3	9	−180	−90	Postcentral L, precentral L, superiorparietal L	136	405	Precentral L, postcentral L, supramarginal L	318	−50	Postcentral L, precentral L, supramarginal L	0741
	P4	5	−205	−60	Inferiorparietal L, supramarginal L, postcentral L, precentral L	60	270	Supramarginal R, inferiorparietal L, supramarginal L, postcentral L, precentral L	180	10	Inferiorparietal L, supramarginal L, superiorparietal L	1831
	Avg AMC		−215	−54	Precentral L, caudalmiddlefrontal L, superiorfrontal L	98	247	Precentral L, postcentral L, supramarginal L supramarginal R	253	−31	Precentral R, caudalmiddlefrontal R	0741
	C1	10	−194	−36	Caudalmiddlefrontal R, precentral R, postcentral R	28	388	Precentral L, postcentral L, caudalmiddlefrontal L	163	71	Postcentral R, superiorparietal R	2011
	C2	9	−70	−15	Caudalmiddlefrontal L, precentral R, postcentral R, superiorparietal R	88	337	Postcentral L, supramarginal L, superiorparietal L	237	−112	Postcentral L, precentral L	0741
	C3	8	−97	−10	Precentral L, postcentral L, caudalmiddlefrontal L, superiorfrontal L	72	424	Precentral L, caudalmiddlefrontal L, postcentral L	165	66	Postcentral L, precentral L	0741
	C4	10	−74	−42	Precentral L, postcentral L	63	136	Precentral L, postcentral L	129	−147	Superiorparietal R, inferiorparietal R	0741
	C5	10	−188	−67	Supramarginal L	54	445	Superiorfrontal L, precentral L, postcentral L	146	−33	Superiorfrontal L, precentral L, supramarginal R	0741
	Avg HCC		−152	11	Supramarginal L, precentral R, superiorfrontal R	54	292	Precentral L, postcentral L	182	20	Precentral L, postcentral L, supramarginal L	0741
	C6	29	33	−60	Superiorparietal L	91	115	Precentral L, postcentral L, superiorparietal L	186	−502	Precentral L, postcentral L, superiorparietal L	0741
	C7	35	−233	−54	Precentral L, postcentral L, superiorparietal L	77	394	Postcentral L, precentral L	239	−165	Postcentral L, superiorparietal L	0741
	C8	38	−5	−200	Precentral L, caudalmiddlefrontal L, superiorfrontal L	90	206	Postcentral L, precentral L	194	−210	Precentral L, postcentral L, superiorparietal L, supramarginal L	0711
	C9	22	−100	−35	NaN	48	212	Precentral L, postcentral L	210	−125	Postcentral L, supramarginal L	0741
	C10	22	−147	−78	Precentral L, postcentral L, superiorparietal L	45	235	Precentral L, postcentral L	205	−88	Precentral L, postcentral L, superiorfrontal L	0741
	Avg HCA		−152	−14	Precentral L, postcentral L, superiorparietal L	86	208	Postcentral L, precentral L	196	−180	Superiorparietal L, supramarginal L, precentral L, postcentral L	0741

#### Patient #2 (P2)—Girl, 8 Years Old

P2 had a pectoralis major transfer to the biceps brachii to restore elbow flexion. The left elbow efficiency of the patient was poor, but the right was satisfactory. See Table 1 for clinical history details.

The left elbow flexion movement was on average 3.2 s and the right was 2.8 s. The left-side movement was associated with a slow wave magnetic field deflection instead of relatively well-shaped MEF associated with right-side movement. The activity source was spread widely across the right precentral and postcentral gyri and involved parts of the left precentral, postcentral, and superior frontal gyri. This dispersion may have indicated that left elbow flection was more difficult for P2 to perform and that she had to recruit some compensatory mechanisms to perform the task. The parts of the pre- and postcentral gyri associated with wrist and even leg control in a normally developed child were activated. The right elbow flexion movement was also subjectively much slower, and the latency of the maximum in MEFI was twice the corresponding component associated with the left-side movement (100 vs. 47 ms). The response peaks were visually well-shaped, and the activity sources were restricted to the left precentral and postcentral gyri in agreement with the homuncular representation of the right arm. Taken together, this could be a sign that the subject was more confident with right elbow flexion and performed the movement at a comfortable pace without complex, impulsive compensatory patterns.

#### Patient #3 (P3)—Boy, 9 Years Old

P3 had no surgery to restore elbow flexion and participated in the MEG study between two steps of wrist contracture elimination on both sides. His right wrist was in a bandage at the time of the experiment. His elbow joint efficiency (both left and right) was poor. See Table 1 for clinical history details.

As evinced by the accelerometer data, the left elbow flexion duration of this patient was 3.1 s and did not cover the full amplitude of movement. The left-side elbow flexion trials were contaminated with high levels of noise. A visually distinguishable peak emerged in the averaged waveforms from the sensors located over the right sensorimotor area 0–100 ms after movement onset. Normally developed subjects and other patients in this study had peaks that could be identified as MEFI. P3 had a peak of −170 fT at 55 ms but later showed several subsequent peaks with greater amplitude up to a latency of 500 ms. The source localization revealed relatively low activation of both the left and right precentral and postcentral gyri, lasting from 20 to 80 ms, as well as activation in the superior frontal gyrus at 260 ms and finally activation at the right precentral, postcentral, and supramarginal gyri around 460 ms after movement onset.

The right elbow flexion movement was on average 1.8 s. Though peaks were visible on the MEF all over the contralateral and ipsilateral hemispheres, the absolute maximum (405 fT) was in the right parietal area with a latency of 136 ms. The source localization revealed a widespread activation in multiple left and right cortical areas at different moments from 30 to 180 ms. At the moment of maximum MEFI (136 ms), the source activity was restricted to the left precentral, postcentral, and supramarginal areas.

Difficulties experienced by P3 in performing the experimental task greatly impacted the accelerometer trajectories, EMF waveforms and sources.

#### Patient #4 (P4)—Boy, 5 Years Old

P4 had a latissimus dorsi transfer to the biceps position earlier in his life (at 1.3 years old) than the other patients. Surgery on the right side was performed at 3 years 8 months and on the left side 2 years 2 months prior to the MEG investigation. Both arms had good elbow joint efficiency. Unlike other patients who participated in this study, P4 had no indication for wrist surgical correction. See Table 1 for clinical history details.

According to the accelerometers, the movements of the hands were very similar: 2.4 and 2.3 s for the left and right side, respectively. In fact, they did not have considerable differences from those demonstrated by healthy control children. There were wide but relatively clear peaks in the ERF waveforms in both the left and right elbow flexion trials. Both left- and right-side movements were associated with maximum peaks in the contralateral hemispheres, with the same latency of 92 and 60 ms from movement onset, respectively. The absolute maximum amplitude in the LH (while performing ride-side action) was 270 fT, and in the RH (while performing left-side action), it was 190 fT. The source activation, however, was not at its maximum at the moment of MEFI (the biggest ERF peak) but was delayed by 20–30 ms. Beamformer analysis showed the maximum PNAI at around 120 ms for the left-side movement trials. Activation was spread among the postcentral, superior parietal, and inferior parietal gyri. For the right-side movement, bilateral activation was evident in multiple cortical areas. At the maximum, around 80 ms, it involved the left and right supramarginal gyri as well as the left inferior parietal, postcentral, and precentral gyri.

Interestingly, in the case of P4, the near-perfect performance was associated with relatively uncertain activation maps on the source-estimation level.

### Healthy Control (Normally Developed) Children (8–10 Years Old), HCC

**Figure 3:**
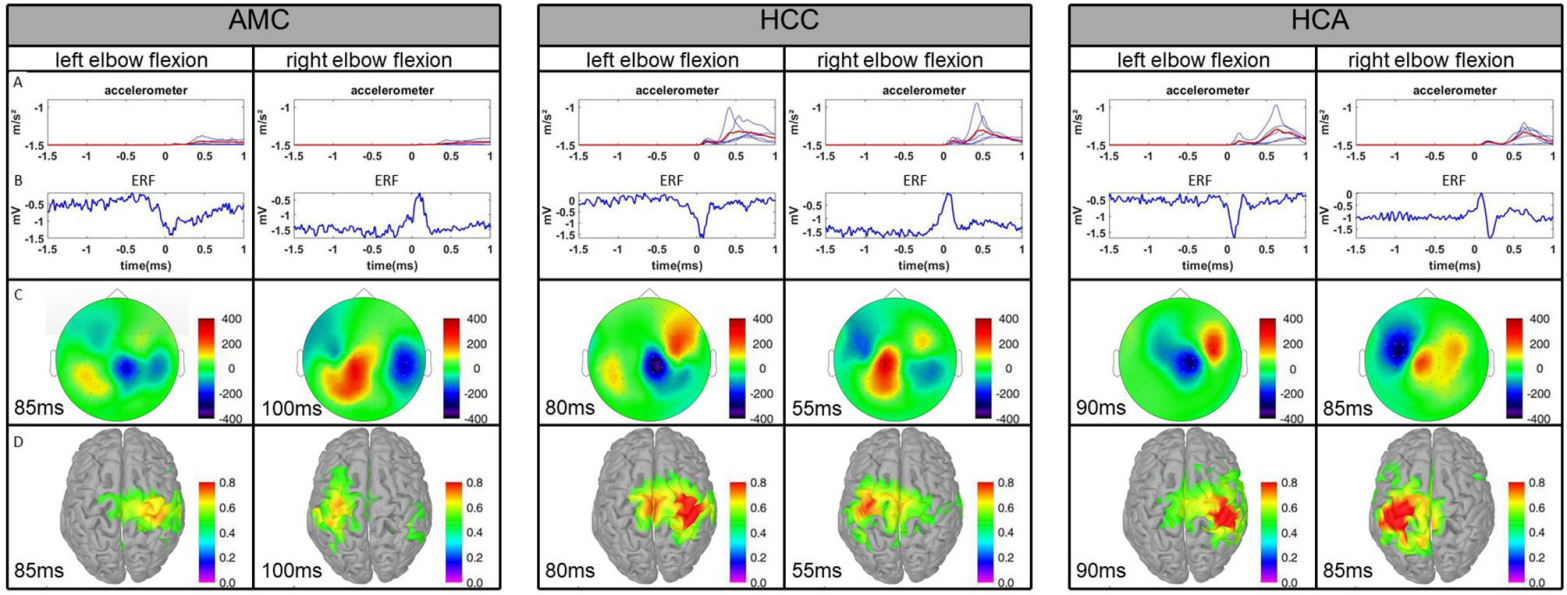
Comparison of the averages of the three experimental groups. AMC, AMC patients; HCC, healthy control children; HCA, healthy control adults. **(A)** Plot of averaged accelerometer power for each subject for the left and right elbows. The red line represents the group average. **(B)** Averaged ERFs at the representative channel. **(C)** Topographies of averaged ERFs at maximum peak latency. Positive values indicate magnetic flux exiting the head (red colors) and negative flux entering the head (blue colors). **(D)** Source reconstruction of ERFs. Neural activity is superimposed on the individual cortex model.

The HCC group average movement duration was 1.7 s for both hands. The average HCC MEG had the highest amplitude of a main peak among the three observed groups (Figure 3). We can probably characterize it as a MEFI. For the RH at MEG0731, the amplitude reached −334 fT 80 ms after movement onset in the average of the left-side movement trials. In the LH at MEG0741, the amplitude reached 292 fT 54 ms after onset of right-side movement. Despite considerable individual differences and high-level artifact contamination in both control children and patients, on a group level, the maximum MEG activity was always contralateral to the movement hemisphere (Figure 3). These MEFI peaks in the left- and right-side trials were subjectively similar, each with a bimodal maximum with one slightly higher peak than the other in different hemispheres. Considering the sample size, these bimodal peaks perhaps did not reflect any underlying physiology but were simply the results of an unequal contribution to the average by control subject #1, who had a shorter latency MEFI peak and a large amplitude difference between the LH and RH.

The estimated source activity on a group level was higher in the RH in left-side movement trials than in the LH in right-side movement trials. The activation maximum was always contralateral to movement. In the RH, it was relatively equally distributed over the precentral and postcentral gyri 50–90 ms after movement onset. This period of maximum activity was preceded by activation of the precentral gyrus from −10 to 30 ms and followed by a subjectively lower activation predominantly over the postcentral gyrus from 110 to 140 ms.

In the LH, the maximum activity was slightly earlier: 40–80 ms after movement onset for the right-side movement trials. However, the left precentral gyrus activation was always greater than the left postcentral. Activity was detected in the left superior frontal (−20 to 20 ms) and left supramarginal (80 to 110 ms) gyri as well. In general, according to the results of visual inspection of the beamformer source localization analysis, the performance of the right elbow flexion task seemed to require less cortical activation than the left.

### Healthy Control Adults (22–38 Years Old), HCA

The HCA group average movement duration was 1.7 s for both hands. The HCA showed the clearest EMF peaks among the three groups, although the MEFI amplitude was the smallest for the left-arm movement and comparable to patients for the right-arm movement. The MF components were found in the latency range of −100 to −10 ms. Four of the five participants had the biggest MEFI peak of 300–450 fT and latency from 60 to 100 ms. One participant had the biggest MEFII, reaching 400 fT. The latency of MEFII was 150–240 ms across the subjects. The source-estimation analysis showed that in the left-side movement trials, activity of the right precentral and partially postcentral gyri started as early as −50 ms relative to movement onset. The maximum involvement of the right precentral, postcentral, and partially supramarginal gyri was evident at 80–90 ms. The activity in these areas slowly diminished up to 140 ms and reappeared with a lesser amplitude around 190–200 ms. In the right-side movement trials, activation was first seen bilaterally in the precentral gyri at −60 ms. Later, at −40 ms, it became greater in the left precentral gyrus than in the right. Maximum activity in the precentral and postcentral left gyri was seen from 80 to 90 ms and diminished by 120 ms. Later in the trial, minor involvement of the left superior parietal, supramarginal, and postcentral gyri was evident from 180 to 210 ms. These dynamics roughly corresponded to the average MF, MEFI, and MEFII peak latencies. For more detailed information on the control children and adults, please see Table 2.

### Estimated Trial Durations

Since the introduced task was not intended to assess the reaction time of the participants and imitate spontaneous self-feeding process, we report trial consistency information in Supplementary Table 2. The average trial duration (movement onset to onset asynchrony) for AMC patients was 8.23 s; for the healthy control children and adults, the duration was 8.20 and 6.0 s, respectively.

### Summary of Results

We found fairly defined patterns of cortical activation in young patients with arthrogryposis and control participants performing semi-self-paced elbow flexion tasks. In general, patients tended to have more bilateral activation for unilateral movements than healthy participants. The ERF amplitudes were greatest in normally developed kids and smallest (except for the MEFII component) in the group of healthy adults. The peaks were subjectively clearer in healthy adults. As the data indicate, the estimated activity sources were considerably more focused in adults and slightly better structured in healthy children than in patients with AMC. The movement trajectory consistency was subjectively greater in healthy children than in patients and was greatest in healthy adults.

Considering the MEFI, a peak that reflects the afferent feedback influence on the somatosensory cortex—in contrast to the MF reflecting efferent motor control—perhaps the AMC patients demonstrated afferent pathways less affected than efferent ones than the healthy controls.

## Discussion

Despite substantial interest and impressive results in the treatment of patients with arthrogryposis in the last few decades, the mechanisms of the cortical organization of motor control in these patients remain mostly unknown. Our research was particularly motivated by the question to what extent the sensorimotor cortical activity in children with inborn absences or severe restrictions of extremity function who develop AMC is different from the normally functioning brains of their healthy peers. To answer this question, we chose an MRI-MEG integrated approach, which is new to the studies of children with AMC.

We compared individual results of the evoked brain activity in response to self-movements in children with AMC with those of age-matched normally developing children and healthy adults. Three of the four patients had muscle transfers to the biceps brachii, and one only had wrist contracture elimination on both sides.

To the best of our knowledge, no studies of proximal motor function using an MEG approach have been reported so far. We specifically designed and tested a paradigm in which the participants flexed their elbows to imitate the self-feeding process to register the brain correlates with which medical doctors can address the efficiency of extremity treatment in patients with AMC. Most motor and sensorimotor findings reported in the literature have been devoted to distal function (14, 22, 23). The disproportionately few brain studies (24) of proximal function are due to the difficulty of monitoring hand-related activity, which (1) causes vast artifactual activity and (2) recruits several proximal muscles that might be difficult to interpret based on the brain signal.

We, however, admit the significant limitations of our paradigm for further applications in cortical mapping of proximal muscle function. In our view, the paradigm is not strictly specific for proximal muscles, but we chose to use an ecological movement that is aimed to be restored by the operation. Of note, several other upper limb muscles are active apart from the biceps. However, this study is the first attempt to create a bridge between the less subjective clinical assessment of arm flexion in the context of huge variability of the pathological conditions of the patients and the more objective measurements of movement-related brain activity using MEG. Moreover, there are currently no uniform standards in the AMC community in assessing the estimation of motor impairment [only general guidelines are available; see (30, 31)].

The premovement MF, MEFI, and MEFII brain responses of healthy adults who served as control participants corroborated the previous results described for voluntary finger movement (21, 22). According to Cheyne et al. the MF peak observed around 50 ms prior to movement onset represents the preparation and initiation of movement, the MEFI observed 40 ms after movement onset reflects proprioceptive and tactile feedback from the muscle, and MEFII registered at 140 ms is probably responsible for fine movement control and adjustment. In our study, the temporal dynamics appeared to be different from those reported for finger movement (21, 22). The average MF latency was ~75 ms before movement onset, MEFI latency was around 90 ms after, and MEFII was 200 ms after in our adult control group. On the one hand, the delay in both EMG and MEF responses could be due to the neurophysiological differences between distal and proximal muscles, that is, the former are finer and quicker than the latter. On the other hand, the differences in response patterns could be explained by the differences in our experimental manipulations. Finger movements usually show less amplitude and more predictable trajectories than do forearm and shoulder movements. In our experiment, the elbow flexion movement was not restricted in amplitude or angle, and although the biceps brachii is expected to be a major contributor to elbow flexion, the movement involves more than one group of muscles. The results of the source analysis of MEG activity also agree well with previous MEG findings (21, 22). The activation of the precentral and partially postcentral gyri of the contralateral-to-movement hemisphere recorded in our study also well-corresponds to the MF component of adult ERF reported by Cheyne et al. (21). A subjectively greater activation of the contralateral postcentral gyrus than of the precentral gyrus associated with MEFI as well as the relatively equal involvement of both at a latency of MEFII components fits the location of MF and MEF sources in the motor cortex, somatosensory cortex, and both the motor and somatosensory cortex, respectively.

Consistent with previously published observations (14, 23), using visual inspection, we found a relatively wider MEFI peak in typically developed children than in adults. However, the MEFI amplitudes of those children were higher than adult MEFIs and their MEFI latencies were shorter. Higher amplitudes in children can be due to the neuronal sources likely being closer to magneto-gradiometers because of the smaller distance between the head surface and cortex. Shorter latencies could be due to the shorter peripheral axonal pathways in children.

Finally, the MEFIIs were less pronounced in healthy children than in adult controls. The sources of neural activity patterns and amplitude were similar in the groups of healthy children and adults, the only difference being the lower neural activity amplitude in the LH during right elbow flexion movement.

Our study reveals noticeable differences in cortical activation during the performance of elbow flexion tasks by patients with AMC and control children and adults.

First, we found subjectively less consistent movement trajectories registered by 3D accelerometer sensors in the patients with AMC than in their healthy peers and the adult control subjects. The patients also performed movements at a noticeably slower pace. Previous studies have shown the interdependence of movement accuracy and lateralized readiness potentials during self-initiated movement tasks (32). Although we had no chance to access the pre-movement readiness field, which is a direct analog of EEG readiness potentials, the observed movement inconsistency is likely associated with general difficulties of movement initiation in patients with AMC.

The second obvious difference at the level of visual inspection between the clinical and healthy participants was the shape of their MEF peaks. The peaks were the widest in the AMC group, moderate in the healthy control children, and smallest in the adult controls. Only P2 and P3 performing right elbow movement showed MEF peaks visually comparable to those of control children. The slower movement initiation and performance in individual trials and the higher intertrial variance both contributed to the smeared shapes of the peaks. In addition, the peak amplitude was the highest in healthy children, moderate in patients with AMC, and the lowest in adults.

While healthy children and adults had visually similar distributions and amplitudes of source activity, in children with AMC, the neural activity indices were much lower and more dispersed across the cortex than in both healthy children and control adults. Combined with the lower ERF amplitude mentioned above, this outcome might reflect a lesser need for cortical neural resource recruitment to perform the fairly simple task of elbow flexion in adults.

Three patients who had biceps restoration operation demonstrated activation in the somatosensory and motor areas in the contralateral-to-movement hemisphere. On the contrary, the patient without biceps function restoration had no observable activation during attempts at left elbow flexion and almost equal amplitudes in contralateral and ipsilateral cortical activation in the right elbow flexion trials. In almost all runs, all the patients and some control group children showed rather poor or entirely undistinguishable MF components. In the patients, this result could be attributed to a general difficulty in movement initiation, which, in turn, should produce a more diffused recruitment of motor neuronal populations and less defined motor responses. In addition to the generally poorer SNR, the audio cue of the command of the assistant might have created interference. The cortical activation of auditory-evoked fields and the orienting response might have disrupted the anticipated shape of MF components.

The peak that might be considered a MEFI was most distinguishable for all but one patient and for all control children. However, the sources of estimated activity were sometimes localized in both motor and somatosensory areas, so we prefer to put the term “MEF” in quotations. Along with MEF components allocated over somatosensory and motor areas, presumably related to arm muscle control, we can see a widespread activation or even unconnected foci of activity in areas corresponding to the fingers, wrist, and shoulders in patients with arthrogryposis. This situation might be explained by the involvement of a complex compensatory mechanism in the performance of the elbow flexion task in patients.

In both sensor and source space, MEFIIs were also not visually identifiable in patients. Subjectively different movement trajectories in the individual trials of patients appeared to completely blur and smear those components from averaged MEG waveforms.

## Limitations and Future Directions

In our experiment, we observed no pre-movement readiness fields (22) across all three groups of participants. The semi-self-paced design of the task left little to no room for this component to develop. Although participants were informed that it was not a reaction time task and that they could initiate the movements at their convenience any time after the command, most subjects demonstrated movement onset 250–500 ms after the vocal cue.

When comparing the MEG recordings of patients and healthy children with those of adults, a poorer SNR was observed. Despite all the attempts of the supervising clinician and MEG assistant personnel, it was impossible to motivate children to abstain from irrelevant movement. Therefore, the SNR seems to impose a major task-unrelated difficulty that prevents researchers from achieving robust MEG mapping at early ages. In the future, a novel and more affordable MEG technique based on optically pumped magnetometers (OPMs) (33) could appear on the market. OPM technology, which neither relies on expensive (superconducting quantum interference devices) SQUID nor depends on the head size of the participant, apparently will offer more movement flexibility and comfort for participants and help with SNR. We see a great potential in the future MEG studies of AMC patients, particularly in the view of developing more affordable and comfortable MEG systems based on optically pumped magnetometers. The accurate brain mapping of brachii muscles might help estimate their individual potentials and choose suitable donor muscles for reconstructing active elbow flexion. After such operations, patients with AMC can often self-feed and are more independent in daily activities.

Even with few trials, the group of healthy adult controls could reproduce a relatively accurate spatiotemporal chain of premovement MFs and MEFs reported for finger movement (21, 22). However, at the very outset, we must admit that the number of trials (40) per type of movement in this paradigm was unacceptably low. The SNR would need to be better for more reliable results. In our case study, we subjectively chose the optimal SNR magnetometers. For the future progress in AMC research, an objective probably automatic procedure should be implemented to effectively select the representative activation in the cued motor task performance. Initially, we were limited to this number of trials and the test run duration by clinical recommendations due to the quick fatigue and relatively low activity of young patients with arthrogryposis.

Unfortunately, the sizes of the pilot groups do not allow a full statistical comparison, but the results allow tentative conclusions. The rather low amplitude of motor responses and the considerable latency jitter in peak motor activity in the patients indicate that their initiation and performance of the movements had shallower recruitment curves for neuronal activity. Therefore, possible rehabilitation strategies might include facilitating motor initiation with non-invasive brain stimulation.

These preliminary results motivate further investigation of neural cortical reorganization and postoperative plasticity in AMC patients, which will be of great value in the attempts to maximize the possible outcomes in health and life quality improvement of children with severe motor deficits, particularly amyoplasia.

Regarding future experimental challenges, first, to observe and evaluate potential abnormalities in the structure and dynamics of related fields properly, it would be reasonable to develop a protocol with either completely voluntary self-paced movement or with considerable delay between the movement initiation command and its execution. However, both options seem non-trivial with young AMC patients. Second, to limit the contamination of the activity of the target muscles by other muscles, it is necessary to use a manipulation assistance device that restricts movement to a certain fixed trajectory and/or amplitude and potentially narrows the possibility of unwanted muscle activity (24, 34). Third, to improve the overall quality of MEG signal recordings without relying on the aforementioned OPM technology, it would be useful to employ the easy solution of adjusting the position of the child in the MEG scanner with individual 3D fitting caps.

With the potential possibility to achieve better quality recordings of MEG activity in AMC patients, it would be of great interest to apply other approaches popular in recent studies such as oscillatory domain analysis (23, 35–43). We expect that it will give researchers opportunities to reach a new horizon in understanding neurodynamics lying behind the motor activity in an AMC patient.

## Conclusions

We demonstrated for the first time the feasibility of MEG mapping of cortical responses associated with proximal muscle activity. We visually identified both pre-movement and movement-evoked activity as manifested in the MF, MEFI, and MEFII MEG responses in children with AMC and compared them to the ERFs of control adults and age-matched healthy children.

The results of visual inspection of the individual patterns of distributed sources of MF and MEF activity revealed the difference between apparently broader and weaker activation in patients in predominantly precentral, postcentral, and supramarginal areas compared with that in healthy children and adult controls.

To conclude, our multicase results indicate the feasibility of MEG monitoring proximal activity in children with AMC. Our pilot data could be interpreted in the light of the hypothesis regarding the specific neuronal recruitment in AMC and call for further investigations on neuronal dynamics in both post- and preoperative patients.

## Data Availability Statement

The raw data supporting the conclusions of this article will be made available by the authors, without undue reservation.

## Ethics Statement

The studies involving human participants were reviewed and approved by Ethical Committee of the National Research University Higher School of Economics. Written informed consent to participate in this study was provided by the participants' legal guardian/next of kin.

## Author Contributions

SG participated in data collection, data analysis, data interpretation, draft of first manuscript, and final edits after receiving feedback from other authors. EB contributed to concept and design of the study, designed illustrative material, and data interpretation. OA performed surgery prior for AMC patients, contributed to concept and design of the study. MN contributed to concept and design of the study, participated in data collection, and data interpretation. VN contributed to concept and design of the study and data interpretation. OM participated in data collection, data analysis. RC participated in data collection. AS supervised the study and contributed to the concept and designed the experiment.

## Funding

This study was supported by a Russian Science Foundation grant (No. 20-68-47038).

## Conflict of Interest

The authors declare that the research was conducted in the absence of any commercial or financial relationships that could be construed as a potential conflict of interest.

## Publisher's Note

All claims expressed in this article are solely those of the authors and do not necessarily represent those of their affiliated organizations, or those of the publisher, the editors and the reviewers. Any product that may be evaluated in this article, or claim that may be made by its manufacturer, is not guaranteed or endorsed by the publisher.
