# Supplementary material for: Feasibility and Challenges of Performing Magnetoencephalography Experiments in Children With Arthrogryposis Multiplex Congenita
Source: Front Pediatr. 2021 Oct 4;9:626734. doi: 10.3389/fped.2021.626734 (PMC8521161; doi:10.3389/fped.2021.626734)
Supplement: Supplementary file 1 [file Table_1.DOCX]

Supplementary Table 1. The Van Heest scale. This scale includes estimation of elbow active flexion, muscle strength, activities of daily living (ADL), and uses adaptive mechanisms for elbow flexion (table push, trunk thrust, or cervical bending).

| Result | Muscle strength | Elbow active flexion | Use in ADL |
| --- | --- | --- | --- |
| Good | 4/5 | >90° | Actively incorporates transfer; rare adaptive mechanisms used |
| Fair | 3/5 | ˂90° | Transfer augments elbow flexion with occasional adaptive mechanisms |
| Poor | 0-2/5 | None | Uses adaptive mechanisms for elbow flexion or significant loss of other ADL function due to tendon transfer |
