# Supplementary material for: Feasibility and Challenges of Performing Magnetoencephalography Experiments in Children With Arthrogryposis Multiplex Congenita
Source: Front Pediatr. 2021 Oct 4;9:626734. doi: 10.3389/fped.2021.626734 (PMC8521161; doi:10.3389/fped.2021.626734)
Supplement: Supplementary file 2 [file Table_2.DOCX]

Supplementary Table 2. Duration values (in seconds) for elbow flexion trials averaged for individual participants and across groups (AMC- AMC patients; HCC- healthy control children; HCA- healthy control adults). The movement duration (Dur) was assessed by means of Accelerometer values. The response onset asynchrony (ROA) was assessed separately for the left and right hands of every participant as the time period between two consequent elbow flexion movement onsets.

| Participant  id | Group | Left hand | | | Right hand | | | Average  left&right | |
| --- | --- | --- | --- | --- | --- | --- | --- | --- | --- |
|  |  | N | Dur | ROA | N | Dur | ROA | Dur | ROA |
| p1 | AMC | 39 | 1.290 | 9.525 | 38 | 2.141 | 7.831 | 1.715 | 8.678 |
| p2 | AMC | 45 | 3.205 | 8.252 | 39 | 2.760 | 7.730 | 2.983 | 7.991 |
| p3 | AMC | 38 | 3.100 | 7.498 | 40 | 1.853 | 7.762 | 2.476 | 7.630 |
| p4 | AMC | 40 | 2.383 | 8.696 | 40 | 2.341 | 8.537 | 2.362 | 8.616 |
| AMC group |  |  | 2.494 | 8.493 |  | 2.274 | 7.965 | **2.384** | **8.229** |
| c1 | HCC | 40 | 2.223 | 6.339 | 46 | 2.787 | 8.989 | 2.505 | 7.664 |
| c2 | HCC | 37 | 1.352 | 7.427 | 40 | 1.451 | 8.475 | 1.401 | 7.951 |
| c3 | HCC | 39 | 1.521 | 6.275 | 42 | 1.385 | 6.466 | 1.453 | 6.370 |
| c4 | HCC | 47 | 1.626 | 8.767 | 42 | 1.353 | 10.969 | 1.490 | 9.868 |
| c5 | HCC | 46 | 1.731 | 9.544 | 41 | 1.562 | 8.713 | 1.646 | 9.128 |
| HCC group |  |  | 1.691 | 7.670 |  | 1.708 | 8.722 | **1.699** | **8.196** |
| c6 | HCA | 42 | 1.418 | 6.647 | 41 | 1.350 | 6.313 | 1.384 | 6.480 |
| c7 | HCA | 40 | 1.779 | 6.107 | 42 | 1.702 | 5.932 | 1.741 | 6.020 |
| c8 | HCA | 42 | 1.496 | 6.686 | 42 | 1.402 | 5.262 | 1.449 | 5.974 |
| c9 | HCA | 41 | 2.164 | 6.552 | 42 | 2.431 | 6.515 | 2.298 | 6.534 |
| c10 | HCA | 39 | 1.649 | 5.269 | 41 | 1.583 | 4.697 | 1.616 | 4.983 |
| HCA group |  |  | 1.704 | 6.252 |  | 1.694 | 5.744 | **1.698** | **5.100** |
