# Supplementary figures and images for: Feasibility and Challenges of Performing Magnetoencephalography Experiments in Children With Arthrogryposis Multiplex Congenita
Source: Front Pediatr. 2021 Oct 4;9:626734. doi: 10.3389/fped.2021.626734 (PMC8521161; doi:10.3389/fped.2021.626734)

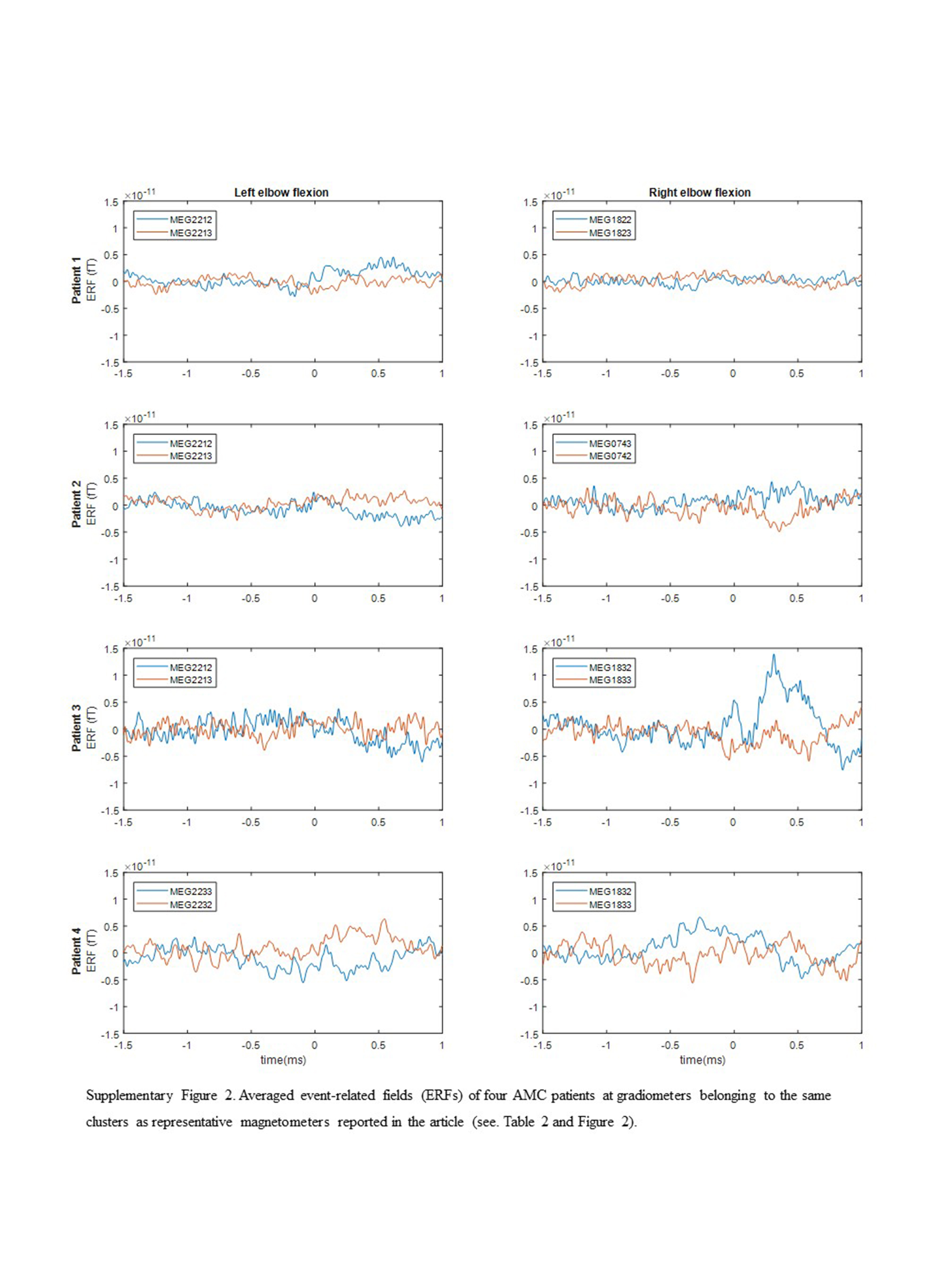

Supplement: Supplementary file 4 [file Image_2.jpg]
